# The International X-Linked Hypophosphatemia (XLH) Registry: first interim analysis of baseline demographic, genetic and clinical data

**DOI:** 10.1186/s13023-023-02882-4

**Published:** 2023-09-27

**Authors:** Gema Ariceta, Signe Sparre Beck-Nielsen, Annemieke M. Boot, Maria Luisa Brandi, Karine Briot, Carmen de Lucas Collantes, Francesco Emma, Sandro Giannini, Dieter Haffner, Richard Keen, Elena Levtchenko, Outi Mӓkitie, M. Zulf Mughal, Ola Nilsson, Dirk Schnabel, Liana Tripto-Shkolnik, Jonathan Liu, Angela Williams, Sue Wood, M. Carola Zillikens

**Affiliations:** 1 Department of Pediatric Nephrology, Hospital Vall d’Hebron, Universitat Autonoma Barcelona, Barcelona, Spain; 2 Centre for Rare Diseases, Aarhus University Hospital, Åarhus, Denmark; 3 Department of Clinical Medicine, Aarhus University, Åarhus, Denmark; 4 Department of Pediatrics, Division of Endocrinology, University Medical Center Groningen, University of Groningen, Groningen, The Netherlands; 5 FIRMO Foundation, Florence, Italy; 6 Donatello Bone Clinic, Florence, Italy; 7 Hôpital Cochin, Service de Rhumatologie, Centre de Référence des Maladies Rares du Métabolisme du Calcium et du Phosphate Filière OSCAR, AP-HP, Paris, France; 8 Universidad Autónoma de Madrid, Madrid, Spain; 9 Hospital Infantili Niño Jesús, Madrid, Spain; 10 Division of Nephrology, Bambino Gesù Children’s Hospital - IRCCS, Rome, Italy; 11 Department of Medicine, Clinica Medica 1, University of Padova, Padua, Italy; 12 Department of Pediatric Kidney, Liver and Metabolic Diseases, Hannover Medical School, Hannover, Germany; 13 Royal National Orthopaedic Hospital, Stanmore, UK; 14 Department of Pediatric Nephrology and Development and Regeneration, University Hospitals Leuven, University of Leuven, Leuven, Belgium; 15 Children’s Hospital, University of Helsinki and Helsinki University Hospital, Helsinki, Finland; 16 Department of Paediatric Endocrinology, Royal Manchester Children’s Hospital, Manchester University Hospital’s NHS Trust, Manchester, UK; 17 Division of Pediatric Endocrinology and Center for Molecular Medicine, Department of Women’s and Children’s Health, Karolinska Institutet and University Hospital, Stockholm, Sweden; 18 School of Medical Sciences and Department of Pediatrics, Örebro University and University Hospital, Örebro, Sweden; 19 Center for Chronically Sick Children, Pediatric Endocrinology, Charité, University Medicine Berlin, Berlin, Germany; 20 Division of Endocrinology, Diabetes and Metabolism, Chaim Sheba Medical Center, Tel Hashomer, Israel; 21 Sackler School of Medicine, Tel Aviv University, Tel Aviv, Israel; 22 Kyowa Kirin International, Marlow, UK; 23 Bone Center, Department of Internal Medicine, Erasmus MC, University Medical Center Rotterdam, Rotterdam, The Netherlands

**Keywords:** X-linked hypophosphatemia (XLH), Hypophosphatemic rickets, Rare disease, International, Natural history, Osteomalacia, Patient registry, *PHEX* mutation, Fibroblast growth factor 23 (FGF23)

## Abstract

**Background:**

X-linked hypophosphatemia (XLH) is a rare, hereditary, progressive, renal phosphate-wasting disorder characterized by a pathological increase in FGF23 concentration and activity. Due to its rarity, diagnosis may be delayed, which can adversely affect outcomes. As a chronic disease resulting in progressive accumulation of musculoskeletal manifestations, it is important to understand the natural history of XLH over the patient’s lifetime and the impact of drug treatments and other interventions. This multicentre, international patient registry (International XLH Registry) was established to address the paucity of these data. Here we present the findings of the first interim analysis of the registry.

**Results:**

The International XLH Registry was initiated in August 2017 and includes participants of all ages diagnosed with XLH, regardless of their treatment and management. At the database lock for this first interim analysis (29 March 2021), 579 participants had entered the registry before 30 November 2020 and are included in the analysis (360 children [62.2%], 217 adults [37.5%] and 2 whose ages were not recorded [0.3%]; 64.2% were female). Family history data were available for 319/345 (92.5%) children and 145/187 (77.5%) adults; 62.1% had biological parents affected by XLH. Genetic testing data were available for 341 (94.7%) children and 203 (93.5%) adults; 370/546 (67.8%) had genetic test results; 331/370 (89.5%) had a confirmed *PHEX* mutation. A notably longer time to diagnosis was observed in adults ≥ 50 years of age (mean [median] duration 9.4 [2.0] years) versus all adults (3.7 [0.1] years) and children (1.0 [0.2] years). Participants presented with normal weight, shorter length or height and elevated body mass index (approximately − 2 and + 2 Z-scores, respectively) versus the general population. Clinical histories were collected for 349 participants (239 children and 110 adults). General data trends for prevalence of bone, dental, renal and joint conditions in all participants were aligned with expectations for a typical population of people with XLH.

**Conclusion:**

The data collected within the International XLH Registry, the largest XLH registry to date, provide substantial information to address the paucity of natural history data, starting with demographic, family history, genetic testing, diagnosis, auxology and baseline data on clinical presentation.

## Background

X-linked hypophosphatemia (XLH) is a rare, hereditary, progressive, lifelong, phosphate-wasting disease resulting in continued accumulation of musculoskeletal manifestations [1]; however, it is still commonly perceived as a pediatric disease [2]. XLH has an estimated incidence of ~ 1 in 20,000–70,000, meeting the European Union definition of a rare disease (< 1 in 2000) [3–7]. In this disease, inactivating mutations in the phosphate regulating endopeptidase X-linked (*PHEX*) gene lead to pathological elevations of the phosphaturic hormone fibroblast growth factor 23 (FGF23) [7]. Excess FGF23 activity leads to increased phosphate excretion by the kidneys and reduced phosphate absorption in the intestines due to low levels of 1,25 dihydroxy vitamin D (calcitriol), impairing bone mineralization and muscle function. In children, this manifests clinically as rickets, short and disproportionate stature, leg bowing, musculoskeletal pain, spontaneous dental abscesses and muscular dysfunction [7, 8]. In adults, continued phosphate wasting and hypophosphatemia leads to osteomalacia, insufficiency fractures and pseudofractures, early onset osteoarthritis and enthesopathy, as well as, for some, hearing loss and spinal stenosis. The early onset osteoarthritis is also thought to be a consequence of bone and joint deformation during growth [1, 9, 10]. All people with XLH, irrespective of age, describe their condition as having a severe negative impact on their physical function, mobility and health-related quality of life [7, 11–14]. ‘Conventional therapy’ for the treatment of XLH includes oral phosphate, given daily in multiple doses, and active vitamin D metabolites or analogues. In contrast to this conventional therapy, the recently licensed anti-FGF23 antibody treatment, burosumab, targets the underlying pathophysiology of XLH by inhibiting and blocking the effects of FGF23, which restores phosphate homeostasis [15–18]. In 2018, burosumab was approved for the treatment of children and adults with XLH by health authorities in the United States and for children with XLH in the European Union. Burosumab was approved for the treatment of XLH in children and adolescents 1–17 years of age with radiographic evidence of bone disease and in adults in the European Union in 2020 [17, 18], and it has recently been included in international clinical practice guidelines as part of the treatment armamentarium [15, 19–21].

Despite XLH being recognized as a chronic, progressive rare disease with a high burden, there is still a paucity of data documenting its natural history and the impact of treatment and other medical interventions on patient outcomes [8]. Disease registries, such as the one described here, can help address this knowledge gap in XLH, as they allow long-term collection of data in the real-world setting. Real-world data are also important to enhance understanding of the natural history of XLH and the impact of alternative treatments, to identify suitable participants to enrol in future clinical trials and studies and to plan healthcare services [22]. Global clinical practice recommendations for the diagnosis and management of XLH call for the development of a comprehensive registry for children and adults with XLH to evaluate the natural history of the disease and the effects of treatment, not only complications [19].

The International XLH Registry is a non-interventional, observational, real-world data collection programme enrolling an intention-to-treat (ITT) population of children and adults with XLH, collecting retrospective and baseline data at registry entry and prospective data during follow-up [8]. This pharmaceutical-sponsored, rare-disease registry, with participating hospital centres across Europe and Israel, collects standard diagnostic and monitoring data, including (where applicable) diagnosis and disease progression history, family history and treatment regimens [8]. With an aim to recruit 1200 participants, and running for 10 years, it is the largest XLH registry worldwide to date. The registry’s aims are to better understand—and improve—life for people with XLH by characterizing disease progression and long-term outcomes associated with XLH and its treatment, enhancing understanding of the natural history and disease burden of XLH, informing the development of future XLH treatment, management guidelines and best practices and describing the effectiveness and safety of treatments used to manage the symptoms and sequelae of XLH [8].

We report here the findings from the first interim analysis of the International XLH Registry, describing the baseline demographic, diagnosis, family history, genetic testing and initial clinical data for 579 children and adults included in this first data cut.

## Methods

### Registry identification

This rare-disease International XLH Registry has been registered with ClinicalTrials.gov, under the identifier NCT03193476 [23]. This registry was initiated in August 2017 and gained its first national regulatory approval in September 2017; this was in the United Kingdom [8], with approval in other countries following shortly after. It aims to include 1200 children and adults with a confirmed diagnosis of XLH and will run for 10 years, at which point the sponsor may decide to continue (or discontinue) the registry, in agreement with the applicable regulatory authorities [8]. The sample size was based on evidence provided by clinical XLH experts across Europe, which suggested that 1200 participants would be sufficient to enable robust research to be conducted on the data collected in the registry. XLH-treating hospital sites underwent a comprehensive screening process to minimise biases and were invited to join the International XLH Registry.

### Registry design

The rationale for and description of the International XLH Registry are described elsewhere [8]. Briefly, the International XLH Registry is an international, multicentre, non-interventional, observational collection of data in a real-world setting. It allows for the capture of clinical outcome variables and treatment details in participants with XLH; participants are followed for as long as informed consent (and assent, where applicable) and regulatory permissions are maintained [8]. Only data collected during standard routine clinical visits and examinations are recorded within the International XLH Registry, and no specific examinations/data entries are mandated, although a core data set has been recommended to participating centres [8]. Any drug therapy considered necessary for the participant’s welfare may be administered at the discretion of the treating physician; all such treatments and any changes that occur throughout participation in the International XLH Registry can be recorded in the database [8].

### Ethics

The International XLH Registry is performed in accordance with the principles set out in the Declaration of Helsinki [24, 25]. The registry protocol was approved at each participating site’s respective country level and/or locally by each site’s ethics committee. Participant information and informed consent were obtained locally from each participant (or their guardian) before inclusion in the International XLH Registry [8].

### Participants in the first interim analysis

The International XLH Registry comprises people of all ages diagnosed with XLH, regardless of their sex, treatment or management [8]. This first interim analysis of the data from the International XLH Registry (database lock: 29 March 2021) includes data from an ITT population who had been entered into the registry prior to 30 November 2020 and who had signed a consent form allowing for their data to be analysed prior to 29 March 2021. At this data cut, participants’ data had been entered from hospital sites in 16 countries: Belgium, Bulgaria, Denmark, France, Germany, Ireland, Israel, Italy, the Netherlands, Norway, Portugal, Slovakia, Slovenia, Spain, Sweden and the United Kingdom. Of note, participant data from Germany was taken from a single centre as part of an existing country-wide Kyowa Kirin International-sponsored investigator-initiated study (IIS). Participants signed a consent form for the IIS which included transfer of patient data to the International XLH Registry.

### Criteria for verifying a diagnosis of XLH

The diagnosis of XLH was based on the clinical judgement of an XLH-treating expert physician, using information such as family history and clinical, radiological and biochemical findings. Dependent on the hospital sites’ accessibility to any available genetic testing, where possible, diagnosis of XLH could also be confirmed by genetic testing (positive for a *PHEX* mutation). As this first interim analysis reports on the full ITT population enrolled in the International XLH Registry, a small number of genetically tested participants with non-*PHEX* mutations (n = 15; 2.6%) were included. Future analyses of the International XLH Registry will remove any participants with non-*PHEX* mutations.

### Data capture and processing

Data capture and data processing included results from genetic testing and other methods of diagnosis, patient demographics, clinical presentation of XLH, length/height, weight and BMI. Analyses of data were descriptive; length/height, weight and BMI were analysed by Z-score (WHO 2007/CDC reference values were used to calculate Z-scores). Data processing was carried out by the contracted research organisation IQVIA (Durham, NC, US). The authors contributed to the statistical analysis plan and interpretation of the data.

## Results

### Registry participant demographics

**Fig. 1:**
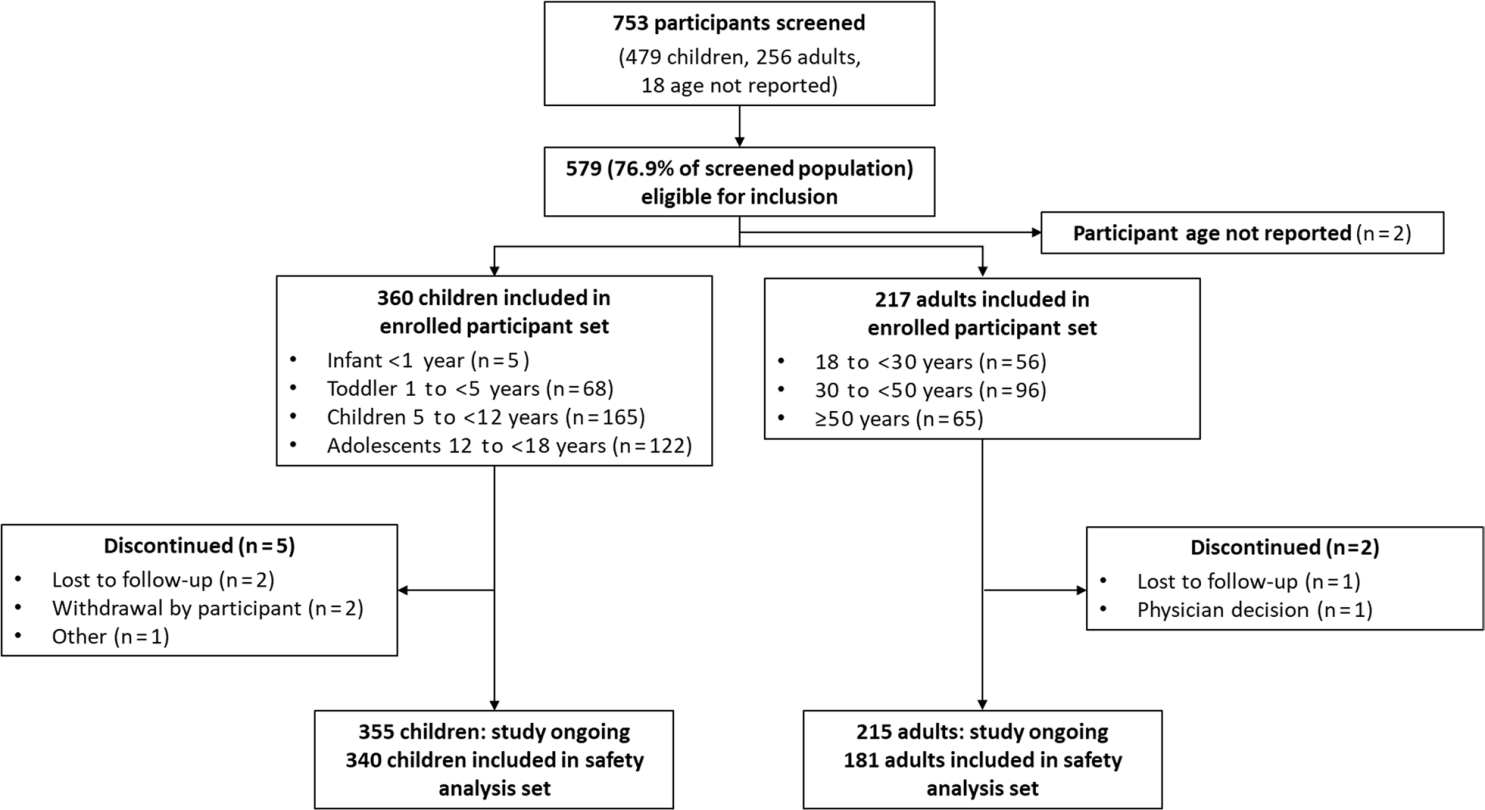
Screening and analysis of participant populations

At the data cut, 753 participants had been screened, of whom 579 (76.9%) were eligible for inclusion in the analysis in accordance with the last date of inclusion (i.e., ‘last patient in’ on 30 November 2020) and the presence of a signed valid consent form at the time of database lock on 29 March 2021 (‘enrolled participants’) (Fig. 1). Participants were from 81 hospital sites in 16 countries. Demographics and baseline characteristics of the enrolled population are presented in Table 1. In total, 374 (64.6%) of this population were female; the population comprised 360 (62.2%) children (< 18 years of age), 217 (37.5%) adults and 2 (0.3%) female participants of unknown age, with a mean (standard deviation [SD]) age at registry enrolment of 9.5 (4.5) years in children and 41.9 (15.5) years in adults. Treatment data at registry entry were available for 401 enrolled participants (281 children, 118 adults, 2 female participants of unknown age). Among the children with available treatment data, 114/281 (40.6%) were receiving conventional therapy (phosphate only, 20/114 [17.5%]; active vitamin D only, 8/114 [7.0%]; phosphate plus active vitamin D, 86/114 [75.4%]), 165/281 (58.7%) were receiving burosumab and 2/281 (0.7%) were recorded as receiving no treatment. Among the adults with available treatment data at the time of registry entry, 99/118 (83.9%) were receiving conventional therapy (phosphate only, 13/99 [13.1%]; active vitamin D only, 25/99 [25.3%]; phosphate plus active vitamin D, 61/99 [61.6%]), 13/118 (11.0%) were receiving burosumab and 6/118 (5.1%) were recorded as receiving no treatment.

**Table 1:** Participant demographics

Characteristic	Participants (N = 579)	
Sex, n (%)		
Female	374 (64.6)	
Male	205 (35.4)	
Age, years, n (%)^a^	(N = 577)	
Pediatric	360 (62.4)	
< 1	5 (0.9)	
1 to < 5	68 (11.8)	
5 to < 12	165 (28.6)	
12 to < 18	122 (21.1)	
Adult	217 (37.6)	
18 to < 30	56 (9.7)	
30 to < 50	96 (16.6)	
≥ 50	65 (11.3)	
Age at enrolment, years, mean (SD) [median]		
Overall	21.7 (18.7) [13.6]	
Pediatric	9.5 (4.5) [9.9]	
Adult	41.9 (15.5) [41.1]	
Race, n (%)^a^	(N = 577)	
White	374 (64.8)	
Black or African American	11 (1.9)	
Asian	6 (1.0)	
American Indian or Alaska Native	1 (0.2)	
Not applicable/not collected as per local regulations	143 (24.8)	
Unknown	27 (4.7)	
Other	15 (2.6)	
Country, n participants enrolled (sites enrolled)		
Belgium	6 (4)	
Bulgaria	0 (2)	
Denmark	12 (5)	
France	141 (7)	
Germany^b^	46 (1)	
Ireland	1 (1)	
Israel	0 (2)	
Italy	36 (10)	
The Netherlands^c^	18 (1)	
Norway	6 (3)	
Portugal	4 (5)	
Slovakia	0 (1)	
Slovenia	2 (1)	
Spain	38 (10)	
Sweden	35 (3)	
United Kingdom	234 (21)	
Treatment distribution at baseline, n (%)^a,d^	Pediatric (N = 360)	Adult (N = 217)
Burosumab	165 (45.8)	13 (6.0)
Conventional therapy only	114 (31.7)	99 (45.6)
Untreated	2 (0.6)	6 (2.8)
Not reported	79 (21.9)	99 (45.6)

N represents number of participants in the main categories: total pediatric participants, total adult participants and total participants. n represents the number of participants within each characteristic

Percentage denominators are the number of participants for whom data was reported in that category

*SD* standard deviation

^a^Age, race and treatment of 2 female participants not captured

^b^Data taken from a single centre as part of an existing country-wide Kyowa Kirin International–sponsored investigator-initiated study

^c^Data taken from a single centre as part of an existing and independent country-wide FGF23-related disease registry

^d^First data entry in the registry

### Genetic testing and diagnosis

In those participants for whom genetic testing and/or diagnosis data were available (n = 546; 341 children, 203 adults, 2 unknown age) (note that the 2 female participants of unknown age are not included in the participant numbers but are included in the total for whom data were available), results of a genetic test were available for 370 (67.8%). Within this group, the proportion of participants with genetic testing results was higher in children (282/341 [82.7%]) than in adults (86/203 [42.4%]). Of those with a genetic test result, most had a confirmed *PHEX* mutation (253/282 [89.7%] children, 76/86 [88.4%] adults). In total, 15 non-*PHEX* mutations were reported: 5 *FGF23* mutations (4 children, 1 adult), 1 *SLC34A3* mutation in a child and 9 ‘other’ mutations (mutation not specified; 7 children, 2 adults). Of those participants tested (n = 368), 24 did not have a confirmed mutation (6.5%). At the time of database lock, 2 results had not been entered into the database.

Where a genetic test was unavailable at a hospital site, in this hereditary X-linked dominant disease, an unambiguous and obvious family history, together with clinical and biochemical manifestations and radiographic imaging, could appropriately confirm a diagnosis of XLH. Family history data were available for 319/345 (92.5%) pediatric participants and 145/187 (77.5%) adult participants. In children, the biological mother was reported to be affected in 164/319 (51.4%); the biological father was affected in 49/317 (15.5%); the mean number of siblings and other family members affected were 0.7 and 1.3, respectively. In adults, 56/140 (40.0%) reported that their biological mother was affected; 22/141 (15.6%) reported that their biological father was affected; the mean number of siblings and other family members affected were 0.7 and 1.3, respectively.

**Fig. 2:**
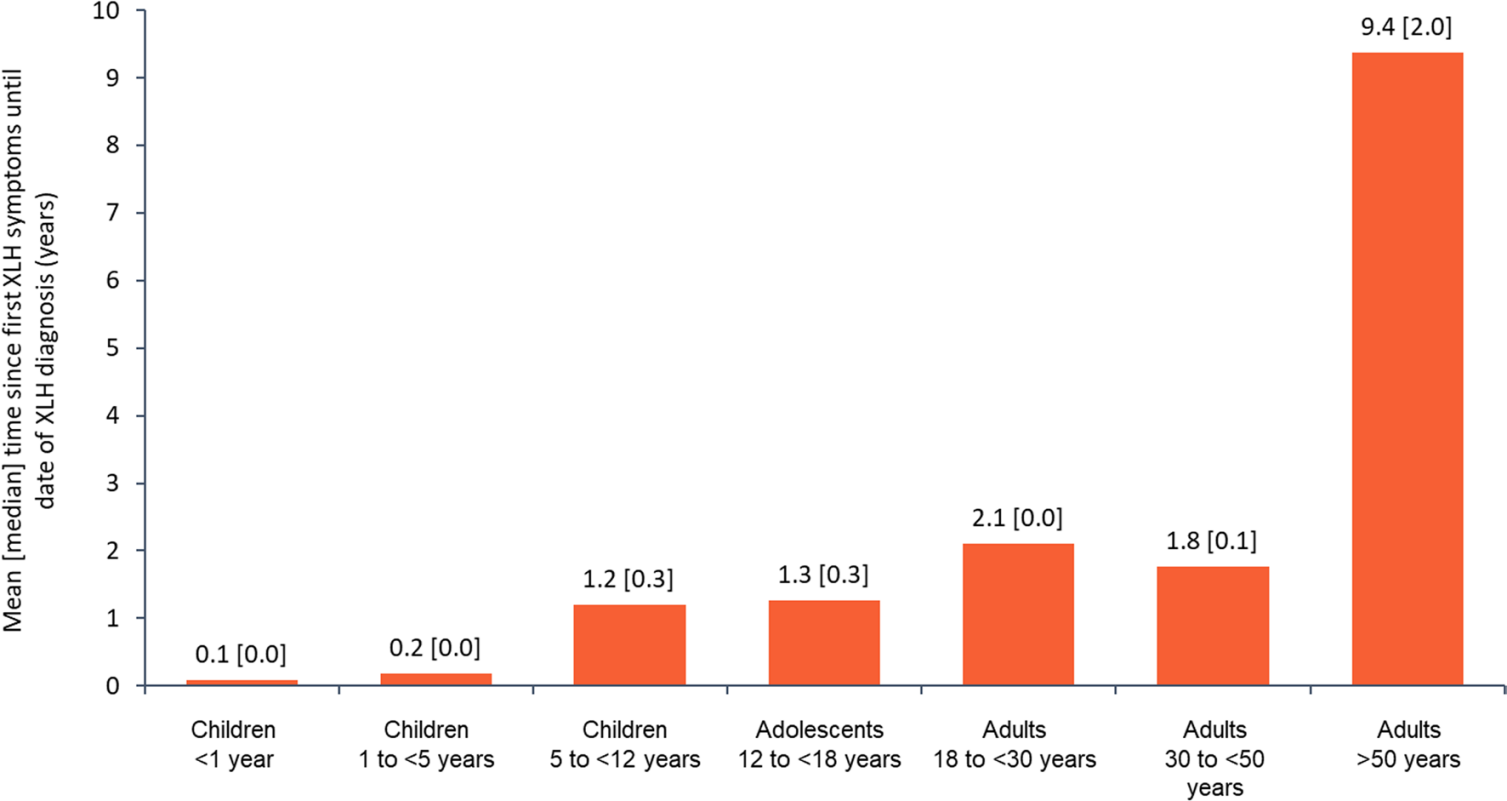
Mean [median] time since first XLH symptoms until date of XLH diagnosis by age group (N = 254). *XLH* X-linked hypophosphatemia

Diagnosis history (i.e., time from first symptoms to diagnosis) was calculable for 254 participants: in children (n = 199), the mean (median) was 1.0 (0.2) years and in adults (n = 55), 3.7 (0.1) years. Adults > 50 years of age (n = 13) had a longer time to diagnosis versus the other adult age cohorts: mean (median) 9.4 (2.0) years (Fig. 2).

### Clinical presentation

Distribution of the different clinical conditions, by body system and age group, are presented in Tables 2, 3, 4 and 5. The diagnosis of these clinical conditions was based on the clinical practice of local expert physicians.

**Table 2:** Retrospective analysis of bone conditions and fractures by age group

Characteristics, n (%)	Children 1 to < 5 years	Children 5 to < 12 years	Adolescents 12 to < 18 years	Total pediatric participants	Adults 18 to < 30 years	Adults 30 to < 50 years	Adults ≥ 50 years	Total adult participants	Total
Bone conditions^a^	n = 33	n = 112	n = 78	N = 223	n = 23	n = 36	n = 20	N = 79	N = 304
Genu varum	27 (81.8)	74 (66.1)	42 (53.8)	143 (64.1)	15 (65.2)	17 (47.2)	10 (50.0)	42 (53.2)	187 (61.5)
Genu valgum	1 (3.0)	47 (42.0)	30 (38.5)	78 (35.0)	9 (39.1)	6 (16.7)	6 (30.0)	21 (26.6)	99 (32.6)
Craniosynostosis	3 (9.1)	24 (21.4)	18 (23.1)	45 (20.2)	0	2 (5.6)	1 (5.0)	3 (3.8)	48 (15.9)
Rachitic rosary	4 (12.1)	13 (11.6)	5 (6.4)	22 (9.9)	2 (8.7)	1 (2.8)	0	3 (3.8)	25 (8.2)
Chiari malformation	3 (9.1)	3 (2.7)	2 (2.6)	8 (3.6)	0	0	0	0	8 (2.6)
Bone fractures	n = 1	n = 3	n = 5	N = 9	n = 4	n = 18	n = 19	N = 41	N = 50
Ribs	0	0	1 (20.0)	1 (11.1)	0	1 (5.6)	2 (10.5)	3 (7.3)	4 (8.0)
Spine	0	0	0	0	0	0	3 (15.8)	3 (7.3)	3 (6.0)
Forearm	0	0	0	0	0	2 (11.1)	0	2 (4.9)	2 (4.0)
Hand/wrist/fingers	1 (100)	0	1 (20.0)	2 (22.2)	0	2 (11.1)	4 (21.1)	6 (14.6)	8 (16.0)
Hips	0	0	0	0	1 (25.0)	1 (5.6)	3 (15.8)	5 (12.2)	5 (10.0)
Femur	0	0	2 (40.0)	2 (22.2)	2 (50.0)	10 (55.6)	10 (52.6)	22 (53.7)	24 (48.0)
Tibia/fibula	0	2 (66.7)	1 (20.0)	3 (33.3)	1 (25.0)	6 (33.3)	4 (21.1)	11 (26.8)	14 (28.0)
Ankle	0	0	0	0	0	2 (11.1)	0	2 (4.9)	2 (4.0)
Feet	0	0	0	0	0	1 (5.6)	1 (5.3)	2 (4.9)	2 (4.0)
Upper arm	0	1 (33.3)	0	1 (11.1)	0	1 (5.6)	0	1 (2.4)	2 (4.0)
Pelvis	0	0	0	0	0	1 (5.6)	0	1 (2.4)	1 (2.0)

N represents number of participants in the main categories: total pediatric participants, total adult participants and total participants. n represents the number of participants in the age groups within the main categories

Percentage denominators are the number of participants for whom data was reported in that category

^a^No bone or fracture data were available for infants (< 1 year of age; n = 5). These items were reported by adults as occurring in their childhood

**Table 3:** Retrospective analysis of dental conditions by age group

Dental conditions, n (%)^a^	Children 1 to < 5 years (n = 6)	Children 5 to < 12 years (n = 54)	Adolescents 12 to < 18 years (n = 36)	Total pediatric participants (N = 96)	Adults 18 to < 30 years (n = 19)	Adults 30 to < 50 years (n = 24)	Adults ≥ 50 years (n = 11)	Total adult participants (N = 54)	Total (N = 150)
Tooth abscess	5 (83.3)	43 (79.6)	29 (80.6)	77 (80.2)	14 (73.7)	19 (79.2)	8 (72.7)	41 (75.9)	118 (78.1)
Excessive cavities	1 (16.7)	21 (38.9)	8 (22.2)	30 (31.3)	7 (36.8)	6 (25.0)	2 (18.2)	15 (27.8)	45 (29.8)
Extraction of adult teeth	0	5 (9.3)	3 (8.3)	8 (8.3)	2 (10.5)	5 (20.8)	5 (45.5)	12 (22.2)	20 (13.2)
Dental implant surgery (to replace missing teeth)	0	0	0	0	0	4 (16.7)	2 (18.2)	6 (11.1)	6 (4.0)
Root canal surgery	0	1 (1.9)	2 (5.6)	3 (3.1)	2 (10.5)	4 (16.7)	5 (45.5)	11 (20.4)	14 (9.3)
Orthodontic treatment	0	3 (5.6)	2 (5.6)	5 (5.2)	0	1 (4.2)	1 (9.1)	2 (3.7)	8 (5.3)
Poor oral health	0	1 (1.9)	0	1 (1.0)	0	0	1 (9.1)	1 (1.9)	2 (1.3)
Enlargement of chamber evocating taurodontism	0	2 (3.7)	0	2 (2.1)	0	1 (4.2)	0	1 (1.9)	3 (2.0)
Presence of radiolucent alveolar bone images	0	0	1 (2.8)	1 (1.0)	0	0	0	0	1 (0.7)
Radiolucent dentine, dentino–enamel junction	0	0	0	0	0	1 (4.2)	0	1 (1.9)	2 (1.3)
Gingivitis	0	1 (1.9)	3 (8.3)	4 (4.2)	1 (5.3)	0	0	1 (1.9)	5 (3.3)
Periodontitis	0	0	0	0	1 (5.3)	4 (16.7)	4 (36.4)	9 (16.7)	9 (6.0)
Oral implant failure	0	0	0	0	0	1 (4.2)	0	1 (1.9)	1 (0.7)
Recurring surgical outcomes	0	0	1 (2.8)	1 (1.0)	0	0	0	0	1 (0.7)

N represents number of participants in the main categories: total pediatric participants, total adult participants and total participants. n represents the number of participants in the age groups within the main categories

Percentage denominators are the number of participants for whom data was reported in that category

^a^No dental data were available for infants (< 1 year; n = 5)

**Table 4:** Retrospective analysis of renal conditions by age group

Renal conditions, n (%)^a^	Children 1 to < 5 years (n = 5)	Children 5 to < 12 years (n = 30)	Adolescents 12 to < 18 years (n = 22)	Total paediatric participants (N = 57)	Adults 18 to < 30 years (n = 4)	Adults 30 to < 50 years (n = 6)	Adults ≥ 50 years (n = 3)	Total adult participants (N = 13)	Total (N = 70)
Nephrolithiasis	0	2 (6.7)	0	2 (3.5)	0	2 (33.3)	1 (33.3)	3 (23.1)	5 (7.1)
Nephrocalcinosis	5 (100)	30 (100)	22 (100)	57 (100)	4 (100)	5 (83.3)	2 (66.7)	11 (84.6)	68 (97.1)

N represents number of participants in the main categories: total pediatric participants, total adult participants and total participants. n represents the number of participants in the age groups within the main categories

Percentage denominators are the number of participants for whom data was reported in that category

^a^No renal conditions data were available for infants (< 1 year; n = 5)

**Table 5:** Retrospective analysis of joint conditions and orthopaedic surgery by age group

Characteristics, n (%)^a^	Children 1 to < 5 years	Children 5 to < 12 years	Adolescents 12 to < 18 years	Total pediatric participants	Adults 18 to < 30 years	Adults 30 to < 50 years	Adults ≥ 50 years	Total adult participants	Total
Joint conditions (osteoarthritis)	n = 2	n = 2	n = 2	N = 6	n = 7	n = 14	n = 19	N = 40	N = 46
Neck	0	0	0	0	0	0	3 (15.8)	3 (7.5)	3 (6.5)
Spine	0	0	1 (50.0)	1 (16.7)	0	3 (21.4)	3 (15.8)	6 (15.0)	7 (15.2)
Shoulder	0	0	0	0	2 (28.6)	2 (14.3)	5 (26.3)	9 (22.5)	9 (19.6)
Wrist	1 (50.0)	1 (50.0)	0	2 (33.3)	1 (14.3)	0	1 (5.3)	2 (5.0)	4 (8.7)
Hip	0	1 (50.0)	0	1 (16.7)	1 (14.3)	5 (35.7)	11 (57.9)	17 (42.5)	18 (39.1)
Knee	1 (50.0)	0	1 (50.0)	2 (33.3)	5 (71.4)	8 (57.1)	11 (57.9)	24 (60.0)	26 (56.5)
Ankle	0	1 (50.0)	0	1 (16.7)	2 (28.6)	0	3 (15.8)	5 (12.5)	6 (13.0)
Foot	0	1 (50.0)	0	1 (16.7)	0	0	0	0	1 (2.2)
Orthopedic surgery	n = 3	n = 18	n = 31	N = 52	n = 26	n = 39	n = 34	N = 99	N = 151
Craniotomy/craniectomy	1 (33.3)	1 (5.6)	6 (19.4)	8 (15.4)	0	1 (2.6)	0	1 (1.0)	9 (6.0)
Stapling of growth plates	0	14 (77.8)	13 (41.9)	27 (51.9)	5 (19.2)	0	2 (5.9)	7 (7.1)	34 (22.5)
Fracture fixation (plates/screws)	0	0	4 (12.9)	4 (7.7)	0	4 (10.3)	3 (8.8)	7 (7.1)	11 (7.3)
Fracture fixation (nails/rods)	0	0	2 (6.5)	2 (3.8)	1 (3.8)	3 (7.7)	4 (11.8)	8 (8.1)	10 (6.6)
Fracture fixation, external	0	0	2 (6.5)	2 (3.8)	2 (7.7)	2 (5.1)	1 (2.9)	5 (5.1)	7 (4.6)
Hip replacement	0	0	0	0	0	4 (10.3)	10 (29.4)	14 (14.1)	14 (9.3)
Knee replacement	0	0	0	0	1 (3.8)	0	8 (23.5)	9 (9.1)	9 (6.0)
Ankle replacement	0	0	0	0	0	1 (2.6)	2 (5.9)	3 (3.0)	3 (2.0)
Osteotomy	2 (66.7)	5 (27.8)	12 (38.7)	19 (36.5)	21 (80.8)	32 (82.1)	17 (50.0)	70 (70.7)	89 (58.9)
Tibial torsion/club foot correction	0	1 (5.6)	1 (3.2)	2 (3.8)	0	0	0	0	2 (1.3)
Leg lengthening	0		2 (6.5)	2 (3.8)	3 (11.5)	2 (5.1)	0	5 (5.1)	7 (4.6)

N represents number of participants in the main categories: total pediatric participants, total adult participants and total participants. n represents the number of participants in the age groups within the main categories

Percentage denominators are the number of participants for whom data was reported in that category

^a^No joint conditions data were available for infants (< 1 year; n = 5)

#### Bone conditions

Skeletal symptoms and/or manifestations (genu varum, genu valgum, craniosynostosis, rachitic rosary or Chiari malformation) were the most frequently reported clinical problems for both the pediatric and adult participants in the registry (Table 2). Of those with data available, skeletal symptoms and/or manifestations were recorded for 223/239 (93.3%) children/adolescents and 79/110 (71.8%) adults. No bone conditions were reported for the infant age group (< 1 year of age); genu varum and genu valgum were the most frequently reported issues across all other age groups. Of those participants reporting bone conditions, genu varum and genu valgum were reported in 143/223 (64.1%) and 78/223 (35.0%) children/adolescents, respectively, and in 42/79 (53.2%) and 21/79 (26.6%) of adults, respectively. After reaching a peak in the 5– < 12 years age group, reported cases of genu varum and genu valgum appeared to decrease with age (Table 2). In adults, enthesopathy was recorded for 17/79 (21.5%) participants with reported skeletal symptoms and/or manifestations. Craniosynostosis was reported in 45/223 (20.2%) children/adolescents with reported skeletal symptoms and/or manifestations.

Historical fracture data (if the participant had experienced a fracture or not) was captured as a separate data field. No differentiation was made in the data collection form between insufficiency or trauma fractures. Data were available for 72 children/adolescents in the registry, of whom 9 were reported to have had a fracture; the others were confirmed as not having had a fracture. Fractures tended to occur in the tibia/fibula, wrist and femur. The data field was completed for 111 adults, of whom 41 were reported to have had a fracture. A femur fracture was declared for 22 adult participants: 2 in the 18–30 years age group and 10 in each of the 30–50 years and ≥ 50 years age groups. Hip fractures were reported for 5 adults.

#### Dental conditions

Dental issues were the second most frequently reported problem in the clinical history of the participants (Table 3). Of the 239 children with available data, 96/239 (40.2%) recorded dental/oral conditions. By far the most frequently reported dental problem was tooth abscess, which was consistently reported by approximately 80% of those with documented dental issues across all pediatric age groups (77/96 [80.2%] in the overall pediatric population). Dental caries (excessive cavities) were reported for 30/96 (31.3%). Children/adolescents ≥ 5 years of age required more intensive interventions, such as extraction of adult teeth (8/96 [8.3%]), orthodontic treatment (5/96 [5.2%]) and root canal surgery (3/96 [3.1%]).

Of the 110 adult participants with available data, 54/110 (49.1%) reported dental/oral conditions; again, dental abscesses were the most frequently reported problem across all adult age groups (41/54 [75.9%]). The need for surgical interventions such as adult tooth extraction, root canal surgery and dental implant surgery increased with age; reports of caries tended to decrease (Table 3).

#### Renal conditions

Renal complications were reported in 57/239 (23.8%) children/adolescents and 13/110 (11.8%) adults (Table 4). In children, 57/57 (100%) participants with renal complications reported nephrocalcinosis and 2 (3.5%) additionally reported nephrolithiasis. In adults, 11/13 (84.6%) participants with renal complications reported nephrocalcinosis and 3/13 (23.1%) reported nephrolithiasis. No other renal conditions or complications were noted.

#### Joint conditions

Joint conditions were rarely reported for those children/adolescents with clinical history data (6/239 [2.5%]); however, the presence of joint problems increased with age in all adults with clinical history data (40/110 [36.4%]) (Table 5). For adults with reported joint problems (N = 40), osteoarthritis of the knees (60.0%), hips (42.5%) and shoulder (22.5%) were the most frequently reported. Presence of osteoarthritis was also reported in the youngest adult age group (18 to < 30 years), with the frequency of reports increasing with age (Table 5).

#### Orthopedic surgery

Details of historical orthopedic surgery were reported for 52/360 (14.4%) children and 99/217 (45.6%) adults (Table 5). In children/adolescents 5 to < 18 years of age, the most common procedures reported included hemiepiphysiodesis or guided growth treatment (stapling of growth plates) (27/52 [51.9%]). Intensive interventions such as osteotomy to correct deformities were also reported (19/52 [36.5%]) in children/adolescents 1 to < 18 years of age. Fracture fixation using plates, rods or external devices was reported for 4, 2 and 2 adolescents (12 to < 18 years of age), respectively. Cranial surgical treatment was noted in 8 children/adolescents 1 to < 18 years of age.

In adults, osteotomy was the most frequent surgical intervention, reported for 70/99 (70.7%) adults. Joint replacements were noted in adults only, with 14 having had hip replacements (4 in the 30 to < 50 years age group, 10 in the ≥ 50 years age group), 9 having had knee replacements (1 in the 18 to < 30 years age group, 8 in the ≥ 50 years age group) and 3 having had ankle replacements (1 in the 30 to < 50 years age group and 2 in the ≥ 50 years age group).

### Auxological data

#### Length/height

**Fig. 3:**
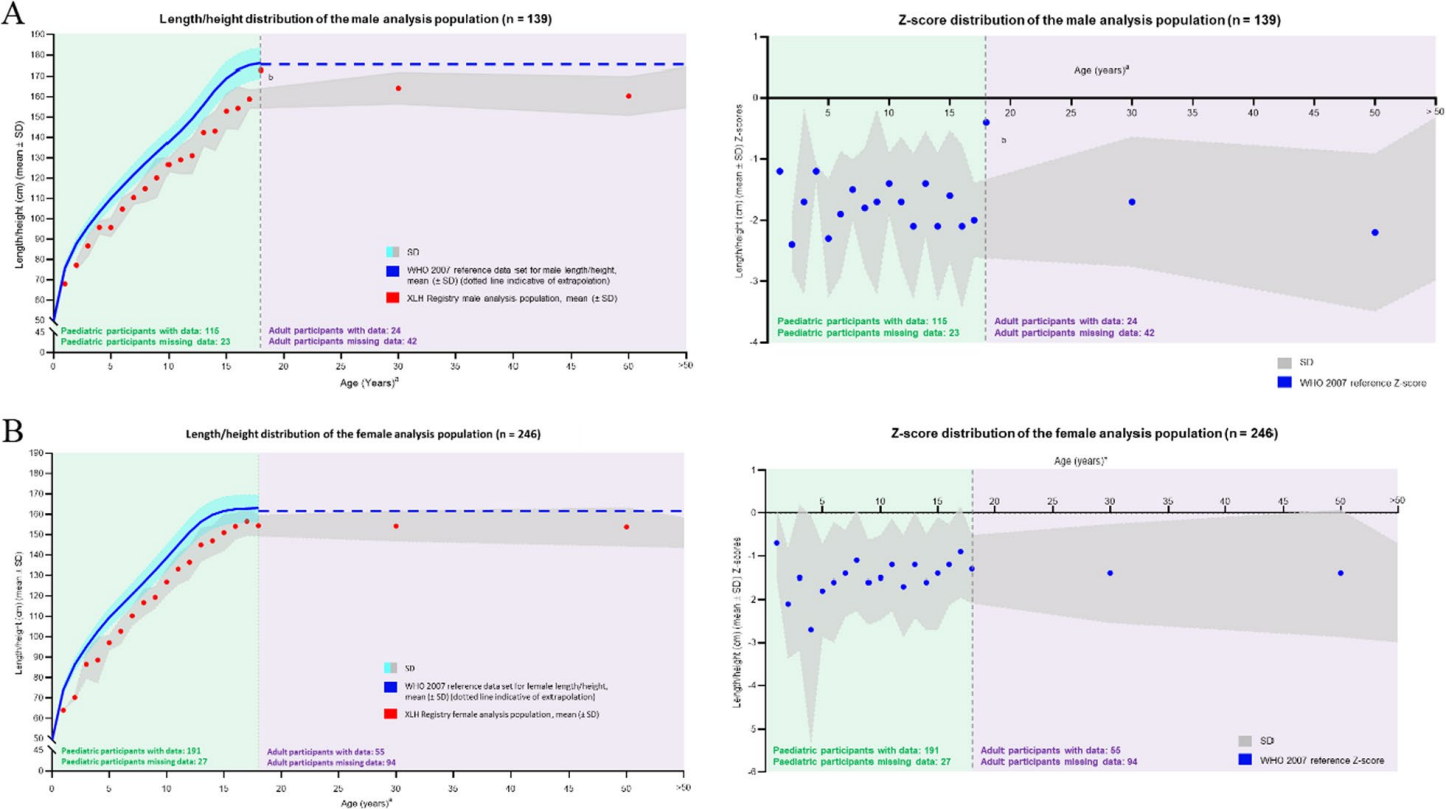
Distribution of length/height standard deviation scores at baseline by age for **A** males and **B** females. ^a^Age (years) is plotted as a range, with each data point plotted at the range max. ^b^Data point is based on 1 participant. Recumbent length used for infants (< 1 year) and toddlers (1 to < 2 years), and standing height used for participants aged ≥ 2 years. *SD* standard deviation; *WHO* World Health Organization; *XLH* X-linked hypophosphatemia

Length (for non-ambulatory infants) and height (for ambulatory children) data were available for 306 children/adolescents (115 boys and 191 girls). Given the quantity of data, average length/height was calculated for each 1-year age tranche from 1 to 18 years to plot growth curves for both males and females in the registry; this was then compared with the yearly average male and female length/height in reference to World Health Organization (WHO) 2007 and Centers for Disease Control and Prevention (CDC) reference values. Z-scores were also calculated at each yearly time point based on the WHO 2007 reference values. Height data were available for 79 adults (24 males and 55 females). Adult data were analysed in a similar way for each of the pre-defined age groups (18 to < 30 years, 30 to < 50 years, ≥ 50 years). Data are shown in Fig. 3a for males and Fig. 3b for females. Absolute length or height tracked consistently below the WHO normal reference values from infancy throughout childhood and in adulthood for both males and females. Z-scores tracked and remained consistent at approximately − 2 from 1 year of age and onwards throughout life.

#### Weight

**Fig. 4:**
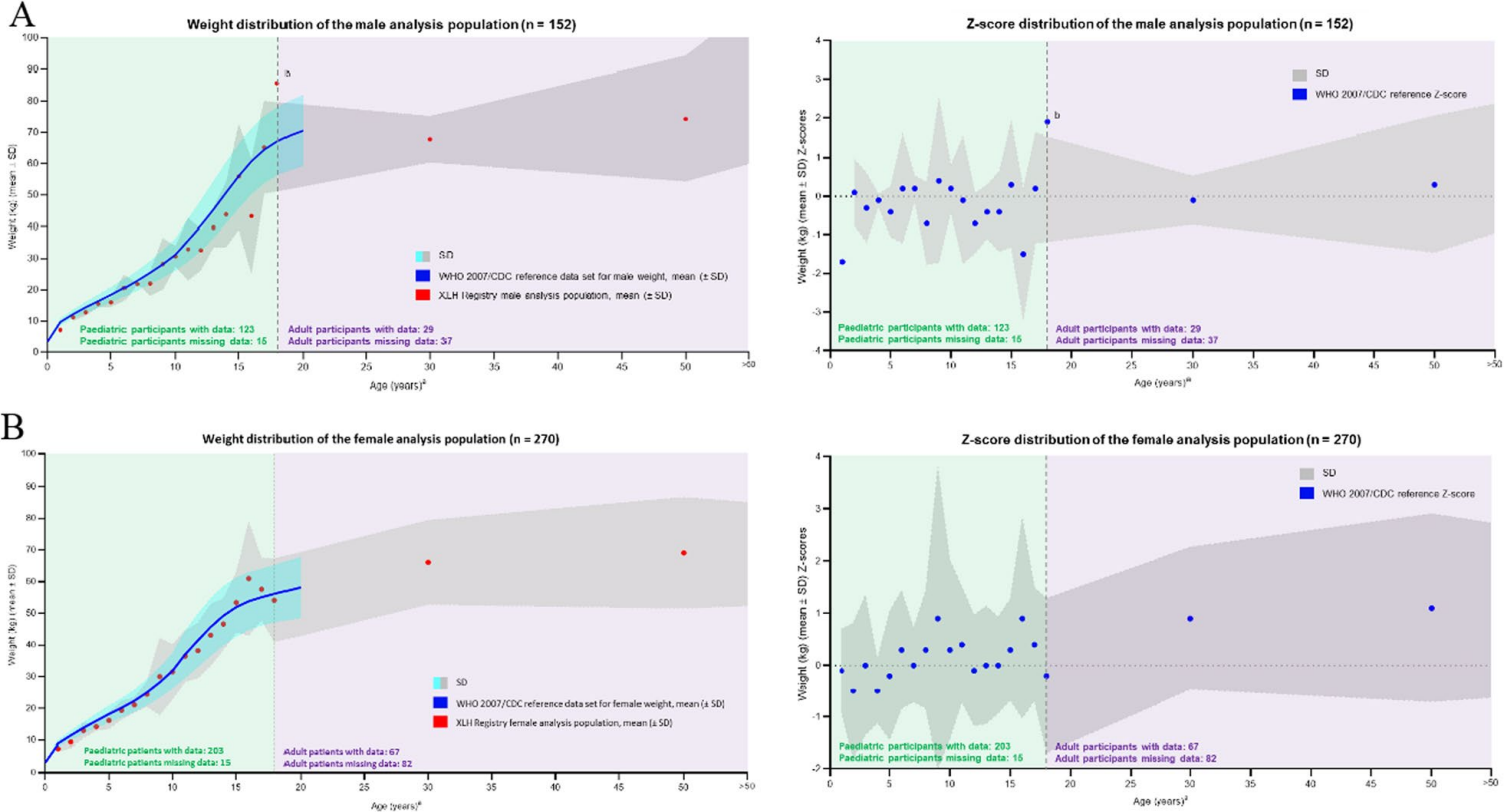
Distribution of weight standard deviation scores by age for **A** males and **B** females. ^a^Age (years) is plotted as a range, with each data point plotted at the range max. ^b^Data point is based on 1 participant. WHO 2007 reference ranges used for ages 1–120 months, and CDC reference ranges used for ages 121–240 months. *CDC* Centers for Disease Control and Prevention, *SD* standard deviation, *WHO* World Health Organization, *XLH* X-linked hypophosphatemia

Weight data were available for 326 children/adolescents (123 boys and 203 girls) and 96 adults (29 males and 67 females). Data were analysed in a similar way to length and height, with average and Z-scores compared with the WHO 2007/CDC reference ranges in 1-year tranches for 1–18 years and for the 3 adult age groups for both males and females. Results and comparison to the WHO 2007/CDC reference values are shown in Fig. 4a for males and Fig. 4b for females. For both sexes (males and females), absolute weight remained very close to the WHO/CDC reference values throughout the lifespan, with the Z-scores also tracking closely to the WHO/CDC reference values for each age point.

#### Body mass index (BMI)

**Fig. 5:**
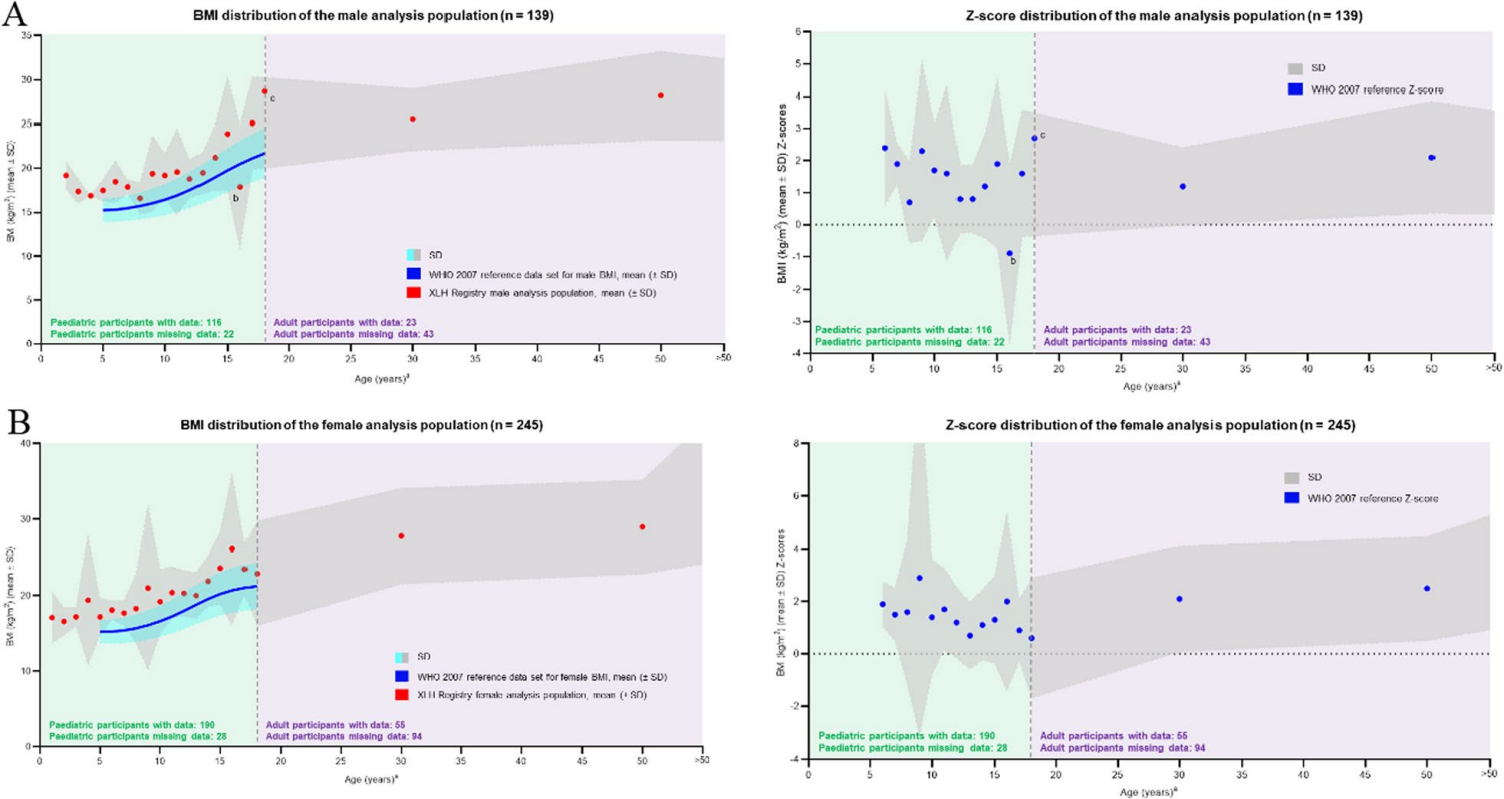
Distribution of BMI standard deviation scores by age for **A** males and **B** females. ^a^Age (years) is plotted as a range, with each data point plotted at the range max. ^b^Data point is based on 7 participants. ^c^Data point is based on 1 participant. *BMI* body mass index, *SD* standard deviation, *WHO* World Health Organization, *XLH* X-linked hypophosphatemia

BMI was calculated for each participant as the ratio of weight divided by length or height (kg/m^2^). BMI data were available for 306 children/adolescents (116 boys and 190 girls) and 78 adults (23 males and 55 females). Average values and Z-scores were calculated in 1-year tranches for children (1–18 years of age) and for the 3 adult age groups and compared with the BMI reference data according to WHO 2007. Results are shown in Fig. 5a for males and Fig. 5b for females. Due to the short stature exhibited across the lifespan in both sexes (male and female) of participants with XLH, together with a relatively normal weight in relation to the WHO/CDC reference, the calculated BMI for all participants, irrespective of age or sex, tracked above the WHO 2007 data set, with Z-scores tracking around the + 2 margin, declining slightly during adolescence and then increasing again in later life.

## Discussion

The demographic and clinical characteristics of the participants of the International XLH Registry data set are consistent with those reported in other XLH studies, case series and non-interventional programmes, all of which supplement the validity and generalizability of these results. Real-world data in rare diseases are essential to help understand the natural history of a disease and recognize treatment effectiveness beyond the efficacy shown in small clinical trials. In this publication of the first interim analysis from the International XLH Registry, the largest in the world reported to date, we describe the results of the analysis of baseline demographic, diagnosis, family history, genetic testing and clinical data. We also establish the validity, credibility and robustness of the registry as a data collection programme and real-world data set. We aim to drive future research questions that can be derived from the data set, such as prospective data trends.

In this International XLH Registry, the sex distribution for XLH was as expected for an X-linked dominant disease, with females comprising over 64% of all participants. As many initial hospital sites were primarily pediatric patient–treating centres, children constituted a major proportion of participants in this interim analysis; subsequent analyses should be more balanced as more adult-treating hospital sites enrol participants. Disease characteristics of adult patients with XLH differ from those of pediatric patients and it will be important to consider these differences in future analyses. Additionally, there may differences in burosumab reimbursement and prescribing practices for differently aged patients. For example, burosumab was initially only approved in patients aged 1 year and older and adolescents with a growing skeleton, and burosumab may be more commonly prescribed in patients with more severe symptoms. These differences will need to be considered in future analyses of this registry.

This first interim analysis of the registry data included a relatively high number of participants from the UK and France (234 and 141, respectively). This could be explained by the higher number of participating sites in those countries, possibly as a consequence of increased centralization of services in those countries, higher hospital site motivation and/or larger patient populations. As this may result in the registry being more representative of clinical practice and burosumab reimbursement in these countries than of all the participating countries as a whole, it will be important to consider differences in clinical practice in the countries included in this registry in future analyses. For example, burosumab reimbursement was not uniformly available, and burosumab treatment may be limited to patients with more severe forms of XLH in some regions. The large data set and number of participating countries and hospital sites in the International XLH Registry make these data more generalizable to larger populations than previous smaller studies. There are many potential explanations for the differences in recruitment between different centres and countries (e.g., ethical board approval, healthcare systems, centralization of care for rare diseases, specific interests and connections of physicians/investigators); Germany, for example, already has its own ongoing pediatric XLH study, which may partially explain why fewer hospital sites in Germany have enrolled in this International XLH Registry.

In this analysis, more children than adults had a genetic test for XLH documented in the data collection form. This result could be partially explained by an increased availability and awareness of the importance of genetic testing in these younger children to confirm the diagnosis, provide an assessment of patient outcome, support treatment decisions and help family genetic counselling. In contrast, there was a relative lack of availability of genetic testing when many older adults were initially diagnosed, and the older population may have been more often diagnosed through clinical, biochemical, radiological and family history. In total, 15 of 579 participants in this first interim analysis of the International XLH Registry had confirmed non-*PHEX* mutations. It must be stressed that the International XLH Registry comprises an ITT population of all enrolled participants with suspected XLH and includes real-world data available at the time of interim analysis. For this current analysis, the percentage of participants with a confirmed non-*PHEX* mutation is small (2.6%) and as such is unlikely to affect the overall results. Future analyses will exclude this patient population.

In this analysis, more children had family history data collected than adults. This may be explained by the increased awareness of the disease and the importance of these data for accurate diagnosis. Family history can be used as a diagnostic tool and to help guide decisions about genetic testing for the participant and at-risk family members. A full genetic pedigree can also capture details about the health of multiple generations. This information can be important in diagnosing an inherited condition such as XLH, revealing the pattern of inheritance and informing clinical decisions regarding genetic testing and management of affected participants.

In this analysis, time to diagnosis from first symptoms was shorter in children and younger adults than in older adults. This may be a consequence of increased knowledge of XLH, improved healthcare systems and access to XLH specialists and a more recent focus on XLH and its genetic origin (*PHEX*). Indeed, current guidelines are aiding earlier diagnosis in younger populations. Of course, further improvements can still be made, for example, the call to encourage routine serum phosphate testing when encountering children with short stature. There is still no general routine set of laboratory tests for the diagnosis of XLH. There is also a lack of consensus regarding therapy in adults [2].

We observed a very high number of skeletal symptoms and manifestations in participants with XLH, even in young adults, which is consistent with other studies of the disease [1–4, 6, 9, 13, 26]. There was a high prevalence of lower limb deformity requiring corrective surgery in children and in adults. Fractures were more common in adults; indeed, large bone fractures (e.g., femur, hip, pelvis) were mainly reported in adults, being present in young adults and generally increasing with age. Femur fractures were most frequent, and hip fractures were reported in adults < 50 years of age and increased with age (> 70 years) [27]. These findings in this International XLH Registry are consistent with previously published data in XLH; Javaid et al. found that 43.3% of adults across all age bands (≥ 18 years) reported a previous fracture, which increased to > 65% in adults > 60 years of age. Femur was the most common fracture type, along with the foot [1]. This compares with 41/111 (36.9%) adults with fractures in this registry; more than half (53.7%) had previous femur fractures. However, a Danish study found a decreased relative risk of fracture among 38 patients with hypophosphatemic rickets compared with controls: 0.34 (95% confidence interval 0.20–0.57, *P* = 0.001) [10].

Orthopedic interventions were reported in children and adults in the International XLH Registry. The most common procedure in children 5 to < 18 years of age was hemiepiphysiodesis, or guided growth therapy, as well as osteotomy to correct deformities. In adults, osteotomy was the most frequent intervention; hip and knee joint replacements were common in adults < 50 years of age, as reported in other studies [1, 10]. Some surgeries are needed to treat unresolved childhood disease [9], with early interventions able to correct or prevent deformities, which helps to improve long-term outcomes. Craniosynostosis was reported in 20.2% of children with reported skeletal symptoms and/or manifestations. In several studies investigating the burden of XLH, craniosynostosis was not reported [1, 9, 28]. However, Skrinar et al. confirmed craniotomy and craniectomy in 3.3% of children and 6.0% of adults surveyed, which may have been performed to treat craniosynostosis, and the presence of Chiari malformations (7.8% in both children and adults) [9], which could have occurred as a result of craniosynostosis [29].

In this registry, dental conditions were frequently reported in children (40.2%) and adults (49.1%) with available data. The need for surgical interventions and dental implant surgery increased with age, while reports of caries actually tended to decrease. A possible explanation for this observation may point to the fact that adult participants may have fewer remaining teeth (due to previous tooth loss/extraction), therefore dental caries would be impossible for non-existent dentition. Dental conditions could also have been under-reported, as dental treatments are typically performed by the participants’ dentist, and their records may not have been available for the physician treating the participant’s general XLH. Indeed, a survey by Nguyen et al. [30] noted that many participants did not receive adequate support for dental conditions and experienced a disorganized care pathway, which may have led to difficulties in collection of patient data. The etiology of dental conditions in XLH is unclear, and the impact of treatment is uncertain. Research in a pre-clinical Hyp (*PHEX* mutant) mouse model revealed that enamel volume was reduced, dentine was malformed and hypomineralized and the alveolar bone was hypomineralized, showing a disorganized structure and increased bone volume [31]. Dental abscesses were a common complaint of children in this registry. This is certainly consistent with the data published by Nguyen et al., who found that dental abscesses were a cause of intense pain, altered smile and problematic chewing—all contributing factors to the reduced quality of life of participants with XLH. This highlights the need for specialized care by dental experts aware of the role of XLH in dental conditions and for early intervention to reduce later complications [30].

Absolute length or height of registry participants tracked consistently below the WHO normal reference values from infancy throughout childhood and into adulthood for both males and females. However, an outlier to this pattern was observed in a single male participant of 18 years of age, whose height approached the WHO reference range. This discrepancy may have been due to the national origin of the participant in question: the Netherlands, a country where the average height of the population is higher compared with the rest of the world. This highlights the differences in growth references between countries [32] and raises an important question about considering standardized versus national measures when analysing international data. This issue will be explored and addressed in future analyses in this registry. Nonetheless, what is clear in all patients with XLH is that longitudinal growth is diminished, with longitudinal growth statistically lower than WHO 2007/CDC reference ranges from ≥ 1 year of age [33].

High BMI has been reported in other studies of populations of patients with XLH. One third of children in an XLH population in France were reported to be overweight or obese [34], and adolescents and adults with XLH in Austria had a mean elevated BMI, with 35.0% reported as being overweight and 30.0% as obese [35]. However, data collected in this registry have shown that children and adults combine shorter stature with ‘normal’ weight, leading to an elevated BMI. Another potential contributor to elevated BMI could be due to the participant’s proportionally shorter lower limbs: since the torso is heavier than the legs, it would be expected that the torso would contribute a greater proportional weight. In addition, XLH has an effect on mobility, fatigue, pain and muscle weakness; this may result in reduced mobility and activity, which could in turn lead to a more sedentary lifestyle. In consideration of all the possible contributory factors, these length/height, weight and BMI findings reinforce the need for more research in patients with XLH to determine whether traditional methods of measuring BMI are appropriate and whether alternative methods could be explored. High BMI may contribute to increased mortality in XLH [26], and it would be relevant to conduct more research into why patients with XLH do develop weight- and obesity-related metabolic complications.

Several XLH registries are ongoing, but, to our knowledge, no results have been published in peer-reviewed journals to date. SUNFLOWER (Japan/South Korea) [36] and the Disease Monitoring Program (United States/Latin America) [37] are industry-sponsored, with others organized by academic/medical societies (e.g., the European Reference Network on Rare Bone Diseases [ERN BOND] [38]). SUNFLOWER is a longitudinal, observational cohort study in 160 Asian participants with XLH (initiated in April 2018, with registration remaining open until 30 April 2022; the planned follow-up is 5 years) [36]. It will be important to compare the results and outcomes of these other ongoing studies with this International XLH Registry of participants of European and Israeli descent.

This registry does have some limitations. As with all observational registries, there were no requirements for mandatory data completion, and some registry data were not yet recorded for some parameters at the time of data cut. Some countries and hospitals did not record all variables as part of their standard clinical practice, and these data, as per the ‘entry’ in the Electronic Data Capture database, would have been recorded as ‘Missing’ data. This highlights the need to refine the Electronic Data Capture database: differentiation is needed to determine the exact nature of data not entered, for example ‘Data not reported yet’ versus ‘Data missing (i.e., lost/irretrievable)’. This ‘Missing’ data is a potential source of bias and could limit the generalizability of the findings; for example, if the data are not missing at random but are due to data availability/data collection practices from individual countries or clinical sites. It will be necessary to report and consider missing data, including any patterns present, when conducting future analyses. Nonetheless, useful conclusions can still be drawn from such a large volume of data.

An additional limitation of the registry is that the diagnosis of clinical conditions is based on the local practice of expert physicians. While this ensures that the registry accurately reflects the real-world treatment of patients with XLH in the participating countries, the lack of central definitions of diseases or diagnosis practices may result in regional differences in diagnostic practice or criteria. This in turn may lead to regional variability in the diagnosis, and therefore, reporting of clinical conditions.

In addition to limitations due to the nature of rare-disease registries, there may be different findings, not only related to XLH itself, but to changes in practice over time; for example, even within the past 2 years, updates in guidance and practice have been published [20, 39], some of which are country-specific [20]. Differences between children and adults with XLH may exist due to changes in therapy, changes in other treatments (such as surgery) and, more recently, a greater awareness of XLH and treatment-related complications.

Although strict inclusion criteria were specified in the International XLH Registry, participants were entered into the database by healthcare professionals in good faith that participants had a confirmed XLH diagnosis. The interim analysis of the International XLH Registry described here includes the ITT population of all participants who signed initial consent forms. A genetic test was not a mandated inclusion criterion. When the data were analysed, we found a small number of participants with confirmed non-*PHEX* mutations. Although we must accept the possibility of misdiagnosis in a real-world setting, the inclusion in the registry of a small number of participants who had a confirmed non-*PHEX* mutation emphasizes the need to perform a full family and genetic history of each affected participant to ensure correct diagnosis. All participants will be followed up as part of the registry (unless they withdraw consent) and will be reported in subsequent analyses of patients with ‘confirmed’ XLH on conventional therapy or burosumab.

The baseline data from such a large data set corroborate other studies and observations [33] and also show that, even with early diagnosis and medical management, children with XLH are still not thriving as they should. Questions do still remain, such as whether high BMI translates into morbidity or shortened survival compared with the general population [40], and how the chronicity of problems affect participants’ life courses, as previous studies have suggested that patients with XLH do have a shortened survival [26]. The long-term data that this registry will supply will hopefully provide more information to help answer these questions, thus will be useful in helping to address the paucity of disease natural history and treatment data in XLH. The ultimate aim of the registry is to provide evidence to support and improve patient outcomes in the long term.

## Conclusions

We describe here the first interim analysis of the baseline data from the International XLH Registry, which holds the largest data set of participants with XLH in the world to date. The demographic and clinical characteristics of this data set are consistent with those reported in other XLH studies, case series and non-interventional programmes, all of which add to the general validity and generalizability of these results. A good proportion of participants have a strong family history and/or genetic diagnosis, especially children. This is a robust data set that will mature and increase over time, growing and extending in terms of numbers, countries, hospital sites and prospective follow-up data. Future analyses will seek to provide new data on the progressive, accumulative nature of this disease as well as new insights describing the impact of the different treatment options for XLH that are now in the current treatment armamentarium.

## Data Availability

The data sets used and analysed to support the findings of this study are not openly available due to reasons of sensitivity. However, they are available from the corresponding author upon reasonable request. All data are in controlled access data storage at IQVIA.

## Abbreviations

BMI: Body mass index
CDC: Centers for Disease Control and Prevention
ERN BOND: European Reference Network on Rare Bone Diseases
FGF23: Fibroblast growth factor 23
ITT: Intention-to-treat
*PHEX*: Phosphate regulating endopeptidase homologue, X-linked
SD: Standard deviation
WHO: World Health Organization
XLH: X-linked hypophosphatemia

## References

[1] Javaid MK, Ward L, Pinedo-Villanueva R, Rylands AJ, Williams A, Insogna K, et al. Musculoskeletal features in adults with X-linked hypophosphatemia: an analysis of clinical trial and survey data. J Clin Endocrinol Metab. 2022;107:e1249–62. 10.1210/clinem/dgab739. 10.1210/clinem/dgab739 PMC8852215 34636401

[2] Giannini S, Bianchi ML, Rendina D, Massoletti P, Lazzerini D, Brandi ML (2021). Burden of disease and clinical targets in adult patients with X-linked hypophosphatemia. A comprehensive review. Osteoporos Int.

[3] Beck-Nielsen SS, Brock-Jacobsen B, Gram J, Brixen K, Jensen TK (2009). Incidence and prevalence of nutritional and hereditary rickets in southern Denmark. Eur J Endocrinol.

[4] Endo I, Fukumoto S, Ozono K, Namba N, Inoue D, Okazaki R (2015). Nationwide survey of fibroblast growth factor 23 (FGF23)-related hypophosphatemic diseases in Japan: prevalence, biochemical data and treatment. Endocr J.

[5] European Union. Rare diseases. https://ec.europa.eu/health/non_communicable_diseases/rare_diseases_en. Accessed Nov 2021.

[6] Rafaelsen S, Johansson S, Raeder H, Bjerknes R (2016). Hereditary hypophosphatemia in Norway: a retrospective population-based study of genotypes, phenotypes, and treatment complications. Eur J Endocrinol.

[7] Beck-Nielsen SS, Mughal Z, Haffner D, Nilsson O, Levtchenko E, Ariceta G (2019). FGF23 and its role in X-linked hypophosphatemia-related morbidity. Orphanet J Rare Dis.

[8] Padidela R, Nilsson O, Makitie O, Beck-Nielsen S, Ariceta G, Schnabel D (2020). The international X-linked hypophosphataemia (XLH) registry (NCT03193476): rationale for and description of an international, observational study. Orphanet J Rare Dis.

[9] Skrinar A, Dvorak-Ewell M, Evins A, Macica C, Linglart A, Imel EA (2019). The lifelong impact of X-linked hypophosphatemia: results from a burden of disease survey. J Endocr Soc.

[10] Beck-Nielsen SS, Brusgaard K, Rasmussen LM, Brixen K, Brock-Jacobsen B, Poulsen MR (2010). Phenotype presentation of hypophosphatemic rickets in adults. Calcif Tissue Int.

[11] Carpenter TO, Imel EA, Holm IA, Jan de Beur SM, Insogna KL (2011). A clinician's guide to X-linked hypophosphatemia. J Bone Miner Res.

[12] Reid IR, Hardy DC, Murphy WA, Teitelbaum SL, Bergfeld MA, Whyte MP (1989). X-linked hypophosphatemia: a clinical, biochemical, and histopathologic assessment of morbidity in adults. Medicine.

[13] Cheung M, Rylands AJ, Williams A, Bailey K, Bubbear J (2021). Patient-reported complications, symptoms, and experiences of living with X-linked hypophosphatemia across the life-course. J Endocr Soc.

[14] Yanes MIL, Diaz-Curiel M, Peris P, Vicente C, Marin S, Ramon-Krauel M (2022). Health-related quality of life of X-linked hypophosphatemia in Spain. Orphanet J Rare Dis.

[15] Saraff V, Nadar R, Hogler W (2020). New developments in the treatment of X-linked hypophosphataemia: implications for clinical management. Paediatr Drugs.

[16] Brener A, Lebenthal Y, Cleper R, Kapusta L, Zeitlin L (2021). Body composition and cardiometabolic health of pediatric patients with X-linked hypophosphatemia (XLH) under burosumab therapy. Ther Adv Endocrinol Metab.

[17] European Medicines Agency. CRYSVITA 10 mg solution for injection. Summary of product characteristics. https://www.ema.europa.eu/en/documents/product-information/crysvita-epar-product-information_en.pdf. Accessed Nov 2021.

[18] US Food and Drug Administration. CRYSVITA (burosumab-twza) injection, for subcutaneous use. Highlights of prescribing information. https://www.accessdata.fda.gov/drugsatfda_docs/label/2019/761068s004lbl.pdf. Accessed Nov 2021.

[19] Haffner D, Emma F, Eastwood DM, Duplan MB, Bacchetta J, Schnabel D (2019). Clinical practice recommendations for the diagnosis and management of X-linked hypophosphataemia. Nat Rev Nephrol.

[20] Laurent MR, De Schepper J, Trouet D, Godefroid N, Boros E, Heinrichs C (2021). Consensus recommendations for the diagnosis and management of X-linked hypophosphatemia in Belgium. Front Endocrinol.

[21] Padidela R, Cheung MS, Saraff V, Dharmaraj P (2020). Clinical guidelines for burosumab in the treatment of XLH in children and adolescents: British paediatric and adolescent bone group recommendations. Endocr Connect.

[22] Viviani L, Zolin A, Mehta A, Olesen HV (2014). The European Cystic Fibrosis Society Patient Registry: valuable lessons learned on how to sustain a disease registry. Orphanet J Rare Dis.

[23] ClinicalTrials.gov. ClinicalTrials.gov Identifier: NCT03193476. Registry for patients with X-linked hypophosphatemia (XLH Registry). https://clinicaltrials.gov/ct2/show/NCT03193476 Accessed Nov 2021.

[24] European Medicines Agency. ICH: E 6 (R2): Guideline for good clinical practice (ICH GCP). https://www.ema.europa.eu/documents/scientific-guideline/ich-e-6-r2-guideline-good-clinical-practice-step-5_en.pdf. Accessed Nov 2021.

[25] International Society for Pharmacoepidemiology. Guidelines for good pharmacoepidemiology practices (GPP). https://www.pharmacoepi.org/resources/policies/guidelines-08027/. Accessed Nov 2021.

[26] Hawley S, Shaw NJ, Delmestri A, Prieto-Alhambra D, Cooper C, Pinedo-Villanueva R, et al. Prevalence and mortality of individuals with X-linked hypophosphatemia: a United Kingdom real-world data analysis. J Clin Endocrinol Metab. 2020;105:e871–8. 10.1210/clinem/dgz203. 10.1210/clinem/dgz203 PMC7025948 31730177

[27] Curtis EM, van der Velde R, Moon RJ, van den Bergh JPW, Geusens P, de Vries F (2016). Epidemiology of fractures in the United Kingdom 1988–2012: variation with age, sex, geography, ethnicity and socioeconomic status. Bone.

[28] Ito N, Kang HG, Nishida Y, Evins A, Skrinar A, Cheong HI (2022). Burden of disease of X-linked hypophosphatemia in Japanese and Korean patients: a cross-sectional survey. Endocr J.

[29] Rothenbuhler A, Fadel N, Debza Y, Bacchetta J, Diallo MT, Adamsbaum C (2019). High incidence of cranial synostosis and Chiari I malformation in children with X-linked hypophosphatemic rickets (XLHR). J Bone Miner Res.

[30] Nguyen C, Celestin E, Chambolle D, Linglart A, Duplan MB, Chaussain C (2022). Oral health-related quality of life in patients with X-linked hypophosphatemia: a qualitative exploration. Endocr Connect.

[31] Zhang H, Chavez MB, Kolli TN, Tan MH, Fong H, Chu EY (2020). Dentoalveolar defects in the *Hyp* mouse model of X-linked hypophosphatemia. J Dent Res.

[32] Bonthuis M, van Stralen KJ, Verrina E, Edefonti A, Molchanova EA, Hokken-Koelega AC (2012). Use of national and international growth charts for studying height in European children: development of up-to-date European height-for-age charts. PLoS ONE.

[33] Mao M, Carpenter TO, Whyte MP, Skrinar A, Chen CY, San Martin J, et al. Growth curves for children with X-linked hypophosphatemia. J Clin Endocrinol Metab. 2020;105:3243–9. 10.1210/clinem/dgaa495. 10.1210/clinem/dgaa495 PMC7448934 32721016

[34] Zhukouskaya VV, Rothenbuhler A, Colao A, Di Somma C, Kamenický P, Trabado S (2020). Increased prevalence of overweight and obesity in children with X-linked hypophosphatemia. Endocr Connect.

[35] Mindler GT, Kranzl A, Stauffer A, Kocijan R, Ganger R, Radler C, et al. Lower limb deformity and gait deviations among adolescents and adults with X-linked hypophosphatemia. Front Endocrinol. 2021;12:754084. 10.3389/fendo.2021.754084. 10.3389/fendo.2021.754084 PMC8503556 34646241

[36] Kubota T, Fukumoto S, Cheong HI, Michigami T, Namba N, Ito N (2020). Long-term outcomes for Asian patients with X-linked hypophosphataemia: rationale and design of the SUNFLOWER longitudinal, observational cohort study. BMJ Open.

[37] ClinicalTrials.gov. ClinicalTrials.gov Identifier: NCT03651505. X-linked hypophosphatemia disease monitoring program. https://www.clinicaltrials.gov/ct2/show/NCT03651505. Accessed Nov 2021.

[38] Javaid MK, Mordenti M, Boarini M, Sangiorgi L, Group EBW, Westerheim I (2021). Patients' priorities and expectations on an EU registry for rare bone and mineral conditions. Orphanet J Rare Dis.

[39] González-Lamuño D, Lorente Rodríguez A, Luis Yanes MI, Marín-Del Barrio S, Martínez Díaz-Guerra G, Peris P (2022). Clinical practice recommendations for the diagnosis and treatment of X-linked hypophosphatemia: a consensus based on the ADAPTE method. Med Clin.

[40] Iyen B, Weng S, Vinogradova Y, Akyea RK, Qureshi N, Kai J (2021). Long-term body mass index changes in overweight and obese adults and the risk of heart failure, cardiovascular disease and mortality: a cohort study of over 260,000 adults in the UK. BMC Public Health.

